# Non-sugar sweeteners and children: the current picture and controversies

**DOI:** 10.3389/fnut.2025.1676373

**Published:** 2025-11-21

**Authors:** Maria João Campos, Liliana J. G. Silva, André M. P. T. Pereira, Angelina Pena

**Affiliations:** LAQV, REQUIMTE, Laboratory of Bromatology and Pharmacognosy, Faculty of Pharmacy, University of Coimbra, Coimbra, Portugal

**Keywords:** non-nutritive sweeteners, children, non-communicable diseases, marketing and labels, health literacy, policies

## Abstract

The global effort to reduce childhood obesity and type 2 diabetes has increased the use of non-sugar sweeteners (NSS) as alternatives to sugar. However, the long-term health effects of NSS in children remain debated, with inconsistent evidence supporting a precautionary approach. This perspective article critically reviews current knowledge, highlights research gaps, and questions the broader context of NSS usage. There is a general consensus that the use of NSS is inadvisable before age 3 and strongly discouraged for those up to age 5. Beyond this age, uncertainties persist, especially regarding the benefits and risks for children who are overweight, obese, or have diabetes. There is an urgent need for robust, long-term studies on NSS exposure in children. Current regulatory measures such as varied labelling, school restrictions, and taxation reflect the lack of a unified scientific consensus and harmonised policies. To effectively protect children’s health, it is essential to establish a global agreement and develop evidence-based guidelines on the use of NSS.

## 1 Introduction

A global nutrition strategy must prioritise healthy diets to improve health and well-being while addressing the increasing prevalence of non-communicable diseases (NCDs). Notably, the majority of early NCD-related deaths are preventable, and the WHO Sustainable Development Goal 3.4 aims to achieve a one-third reduction in premature NCD-related deaths by 2030 (1).

Diet is one of the primary determinants of an individual’s future health (2). Establishing healthy eating habits early in life can significantly reduce the risk of diet-related chronic diseases. Nowadays, children face growing threats from unhealthy diets and lifestyles. In 2022, 37 million children under the age of 5 and over 160 million children aged 5–19 years were overweight or obese (3, 4).

In 2023, the Declaration of Zagreb emphasised the importance of raising awareness about obesity and its impact on children and future generations (5). The International Diabetes Federation (IDF) estimates that 592 million adults will have type 2 diabetes by 2035. In Europe, the prevalence of this condition is expected to rise by 13% by 2045. Once linked to older adults, type 2 diabetes is increasingly affecting younger populations due to rising obesity rates (6).

Overweight, obesity, and related NCDs are major but preventable public health challenges. Early intervention during childhood can reduce the risk of chronic disease later in life. To safeguard future generations, the WHO aimed to halt the rise in obesity and diabetes by 2025 (SDG 3.4) (1).

The 2015 WHO guideline (7) conditionally recommended limiting free sugars to less than 10% of daily energy intake to reduce the risk of obesity and tooth decay, noting that the certainty of evidence was insufficient to support a strong recommendation. In 2022, the European Food Safety Authority (EFSA) assessment reinforced this advice and supported the use of non-sugar sweeteners (NSS), including for children (8).

In the European Union (EU), sales data from 2016 to 2021 showed a rise in NSS soft drinks, alongside a decline in sugar-sweetened beverages (SSB) (9). The Food and Agriculture Organization (FAO) predicts that by 2032, NNS total consumption will increase by 12% (10). Therefore, it is imperative to recognise the influence of this trend on human health, encompassing a broad spectrum of health outcomes, particularly regarding children (11).

NSS remain controversial due to unclear health and safety effects. While they may reduce calorie intake and improve glycaemic control in individuals with diabetes or obesity, especially in children, their use should be part of a balanced dietary approach (12). Long-term use of NSS has been linked to nervous system effects, metabolic risks, gut microbiota alterations, inflammation, and an increased risk of type 2 diabetes, cardiovascular disease, and mortality (13). NSS can transfer to foetuses and infants through amniotic fluid or breast milk, with some studies suggesting links to preterm birth, allergies, or poorer cognition, although the evidence remains inconsistent (14–16).

Establishing clear links between NSS exposure and disease is difficult due to study limitations and differing views among health authorities and researchers. Further research, including longitudinal studies, randomised controlled trials, and meta-analyses with robust methods and larger samples, is essential to clarify the safety and health effects of NSS. While the EFSA and the Food and Drug Administration (FDA) classify NSS as safe, there continues to be debate and controversy about their health effects.

In 2023, the International Agency for Research on Cancer (IARC) classified aspartame as possibly carcinogenic to humans (Group 2B). However, the EFSA and JECFA reaffirmed the acceptable daily intake (ADI) of 40 mg/kg body weight. While the EFSA and JECFA assess risk and the IARC assesses hazard, all organisations agree that current data show bias and inconsistencies, warranting further research (17).

However, given the uncertainties regarding the long-term safety of NSS in children and the lack of scientific studies, can we safely recommend their use as part of a healthy diet for children? Infants and children face higher exposure relative to body weight and are more vulnerable due to their immature biological systems. Applying the precautionary principle, the WHO issued new guidance in May 2023, advising caution in the use of NSS for preventing weight gain and diet-related NCDs in individuals without diabetes (18). The WHO advises caution against the use of NSS for weight control or NCD risk reduction, citing studies that suggest an increased risk of type 2 diabetes and cardiovascular diseases.

Nevertheless, some authors critique this WHO guideline, highlighting inconsistencies in NSS research due to varied study designs and outcomes. While acknowledging limitations and the need for further studies (16, 19), there is a consensus that artificially sweetened beverages are inadequate substitutes for sugar-sweetened drinks in diabetes prevention (20).

As scientific evidence on the health effects of NSS in children remains inconclusive (12), preventing their exposure is advisable under the European precautionary principle, as exemplified by the European Union’s Regulation 178/2002 (21). Early childhood is a critical developmental period, and acknowledging children’s vulnerability is essential for protective risk assessment and disease prevention. Consequently, to protect children’s health, NSS are prohibited in foods for infants (under 12 months old) and young children (1–3 years old) (22), including dietary foods for those who are not in good health, except where otherwise specified (23).

No long-term studies exist on the effects of NSS in children. Moreover, due to higher intake relative to body weight, especially among overweight, obese, and diabetic children, the likelihood of exceeding the acceptable daily intake (ADI) is found to be elevated in these vulnerable groups. This perspective article provides a comprehensive overview of current knowledge, highlights areas requiring further research, and raises questions regarding the broader “360-degree” context.

## 2 Past and present recommendations

Given the widespread consensus regarding the lack of scientific studies on the long-term health effects of NSS consumption in children, some guidance and precautionary measures have been recommended to protect future generations (16).

The EU Food-Based Dietary Guidelines (FBDG) recommend reducing sweetener intake to prevent metabolic issues (24). The Horizon 2020 SWEET project, presented in 2024, found that low- or no-calorie sweeteners provided modest weight loss maintenance benefits over 1 year, with no significant effects on glucose regulation or cardiometabolic markers. However, its observational data analysis showed inconsistent links between NSS consumption and NCD risk, with results limited by potential bias, residual confounding, and reverse causality (25).

The European Society for Paediatric Gastroenterology, Hepatology and Nutrition (ESPGHAN) advises caution regarding the use of NSS in children’s diets, particularly for children under the age of 3 (26), and the European Academy of Paediatrics (EAP) advises caution regarding the consumption of sugar-sweetened beverages in children (27).

Canada’s Food Guide recommends that all population groups should restrict NSS consumption (28). Furthermore, Health Canada advises that children from birth to 24 months avoid consuming foods or beverages containing NNS (29).

In the United States of America (USA), the 2020–2025 Dietary Guidelines for Americans and the American Diabetes Association recommend that NSS may be acceptable sugar substitutes when consumed in moderation; however, they advise against long-term use (30). Johns Hopkins’ Obesity Management Guidelines do not include NSS among recommended strategies (31). The National Academy of Sciences notes inconsistent recommendations regarding NSS intake for children. Health organisations such as the US Institute of Medicine and the American Academy of Paediatrics advise against promoting NSS consumption in children until more research is conducted to clarify the potential health effects (32).

In 2019, the American Academy of Pediatric Dentistry (AAPD), alongside the Academy of Nutrition and Dietetics (AND), the American Academy of Pediatrics (AAP), and the American Heart Association (AHA), advised against giving NSS-containing beverages to children under five, highlighting the importance of promoting optimal health amidst limited evidence regarding long-term effects of NSS on young children (33).

The Brazilian Ministry of Health advises against NSS consumption in children due to limited evidence (15). Similarly, the US Agriculture Department (2020–2025 Dietary Guidelines) and Uruguay’s Ministry of Health recommend avoiding NSS during the first 2–3 years of life (30, 34).

## 3 Children’s exposure to NSS

Regulatory bodies establish the ADI based on the no-observed-adverse-effect-level (NOAEL), which is the highest intake level of a substance at which no adverse effects are observed in animal studies. A margin of safety is then applied, typically a factor of 100, to account for species differences and individual human variability, resulting in the ADI. Exceeding this threshold, particularly in vulnerable populations such as children who have higher intake relative to their body weight, significantly reduces this safety margin. This is concerning, as it increases the risk of adverse health outcomes, including metabolic, neurological, and gut microbiota alterations, which are not fully understood in the long term for this age group (35).

Exposure assessments for children and adolescents must be updated using current consumption data to accurately evaluate the intake of NSS. It is essential to assess the recent dietary intake of individual NSS across various scenarios and groups. High-risk children, such as those with phenylketonuria (PKU), diabetes, or obesity, have been found to exceed the ADI for acesulfame-K, cyclamate, and steviol glycosides (36).

Several scientific studies from different countries support this evidence. As stated by Walton and Wittekind, high soft drink consumption among infants and toddlers (<4 years) in the WHO European Region leads to high NSS exposure (9).

A 2019 survey in Portugal found that 85% of soft drinks contained various NSS combinations. For children under 8, drinking two 330 mL cans daily could lead to 72.5 and 60% of the ADI for cyclamate and saccharin, respectively. Daily consumption of NSS-containing foods may exceed the ADI, posing a potential risk (37). The German Federal Institute for Risk Assessment (BfR) analysed 92 popular low-calorie or no-added-sugar soft drinks and found high concentrations of NSS, including acesulfame K, aspartame, cyclamate, saccharin, sucralose, and steviol glycosides. Notably, 87 of the beverages contained multiple NSS, according to findings from the BfR MEAL study (38). A survey conducted across the United Kingdom revealed that approximately two-thirds of children consume NSS products, and this trend remained consistent throughout the 11 years of the study (39). In Chile (2020), NSS were found in nearly all powder juices (98.8%), flavoured milks (98.3%), jellies (91.2%), and dairy desserts (79%) targeted at children, with few NSS-free options, especially for lower-weight children at the risk of exceeding ADI (40). Similarly, a 2024 survey confirmed high NNS consumption among Chilean toddlers, with sucralose being the most prevalent sweetener (41, 42).

## 4 What is being done?

The European Commission’s Health Promotion and Disease Prevention Knowledge Gateway supports policies to regulate NSS intake (43). Countries have adopted measures such as restrictions, incentives, taxation, labelling, and public health campaigns. For effective impact, these interventions should be simple to implement and scalable to shift NSS consumption patterns (44, 45).

### 4.1 Marketing and labels

The global food and soft drink industry spent over 33 billion USD on advertising in 2020, highlighting the power of marketing strategies. Research shows that marketing strongly influences children’s food choices and consumption, contributing to sector growth. This influence has been associated with various forms of malnutrition, including childhood obesity driven by overconsumption (46).

Food marketing and product labelling aimed at children must be carefully regulated to protect their health, aligning with nutritional guidelines such as those from the WHO to prevent diet-related chronic diseases. While nutritional labels list the presence of NSS in ingredients, they typically do not disclose the specific quantities used. Regulations on food marketing and labelling for children differ across countries, reflecting varying public health priorities and consumer protection strategies. Ensuring clear, consistent labelling and responsible marketing is essential to support healthier dietary choices among children.

The AAP recommends that product labels specify the amount of NSS. This aims to improve NSS monitoring, since these sweeteners are widely available and heavily consumed by children (15). Conversely, the EU has not issued comparable guidance.

In Norway, food products containing NSS are not allowed to be labelled with the keyhole symbol. This front-of-pack nutrition label helps consumers identify healthier food options within a product category (47).

In Chile, packaged foods are required to list specific NSS and their quantities (44). Mexico was the first to mandate a warning label advising against NSS for children (45). Both Mexico and Argentina use front-of-package warnings—e.g., “Contains sweeteners, not recommended for children”—to discourage NSS consumption among children.

Children are a growing target in the food market, yet many caregivers and health professionals remain unaware that “reduced sugar” products often contain NSS.

Ambiguous labels such as “No Added Sugar,” “Low Sugar,” “Sugar Free,” or “Diet/Light” may result in inadvertent consumption, thereby underscoring the necessity for clearer, more transparent, and standardised labelling. Consumers possess the right to receive accurate and honest information to help them make informed decisions.

### 4.2 Restricting or eliminating choice

School food policies enforce strict marketing restrictions on food and drinks aimed at children, improving their diets by enhancing nutritional quality. In the EU, along with Norway and Switzerland, several countries have implemented such restrictions, prohibiting the marketing of non-compliant foods and beverages in schools (48).

Greece prohibits NSS in certain beverages in junior high and high school canteens. Hungary bans artificially sweetened soft drinks for children under six in educational settings. Latvia restricts soft drinks with additives, including NSS, except during lunch. Malta and Spain forbid NSS entirely in schools. Wales, UK, takes it a step further by banning all fizzy soft drinks, including diet versions, in primary and secondary schools (46).

A 2018 meta-analysis across multiple countries confirmed that restricting sweetened beverages in schools leads to significant decreases in sugar-sweetened drink and unhealthy snack consumption, without compensatory calorie increases. These findings support the effectiveness of school-level beverage restrictions in reducing both sugar and sweetener intake. The study also noted that decreased sweetened beverage consumption may indirectly reduce exposure to both sugar and NSS, depending on national regulations (49).

### 4.3 Taxes

SSB taxes, based on the amount of sugar, have been implemented in more than 50 countries (50) to encourage the consumption of healthy alternatives, such as beverages with NSS.

A systematic review and meta-analysis of globally implemented SSB taxes indicated a correlation with a decrease in sales of approximately 30%. The most significant decline in SSB sales is attributed to higher taxes, suggesting that prices influence consumer behaviour (51). This measure decreases sugar consumption and reduces the risk of diet-related chronic diseases, and it can be easily implemented for NSS worldwide.

A recent Polish study found that the tax on sweetened beverages prompted many young people to reduce sugary drink consumption, although some switched to artificially sweetened alternatives. While the tax raised awareness and influenced behaviour, additional policies are required to enhance its effectiveness (52).

Spain’s tax on sugar-sweetened beverages reduced consumption mainly among higher-income and health-conscious groups, with limited impact on lower-income populations. The study suggests that while SSB taxes can change drinking habits, targeted measures are needed to address inequalities and improve effectiveness across all groups (53).

In the EU, countries such as France, Spain, Belgium, Norway, and Poland have introduced volume-based taxes on beverages containing NSS (54). It is essential to evaluate the impact and effectiveness of these policies to identify successful strategies for implementing NSS beverage taxes across all EU member states.

## 5 What still needs to be done?

Food preferences develop between the ages of 2 and 3 and are influenced by exposure to processed foods and marketing. Persuasive marketing often promotes unhealthy products, undermining children’s right to health and protection from exploitation. Although parents recognise their role in guiding their children’s diet, many feel powerless against pervasive marketing (55). Despite clear evidence of harm, regulatory actions are limited, while digital marketing expands. Furthermore, industry pressure often obstructs the development and enforcement of effective policies aimed at protecting children’s health.

Notwithstanding recent progress in identifying sustainable and healthful diets for children and teenagers, there are still critical gaps in NSS exposure assessment.

It is recognised worldwide that there is an urgent need for high-quality research, including well-designed experimental studies and rigorous analyses, to monitor long-term NSS exposure and its effects on children’s health. Special attention should be given to the age at initial exposure and to high-risk populations, such as children with obesity and diabetes. Continuous monitoring of NSS consumption is essential to comprehend its health implications.

Moreover, establishing consensus and implementing more rigorous regulations are essential for safeguarding children’s health, based on robust scientific evidence supported by experts and authorities.

The EU should review case studies on NSS policy outcomes in beverages to guide future regulations across member states. Additionally, clear labelling of the type and amount of each NSS in foods and drinks marketed to children should be mandatory across the EU.

Furthermore, precise and accurate label information is crucial in helping parents, caregivers and healthcare professionals understand the composition of products, easily identify the presence of NSS, and make informed, healthier choices for children (56).

Health authorities, academic institutions, and official entities must reach a consensus, grounded in rigorous scientific research, concerning the safety and usage guidelines of NSS.

## 6 Discussion and conclusion

A review of the current scientific literature suggests that evidence on NSS and health outcomes remains inconsistent, particularly in children. While some studies suggest potential links to metabolic risks and altered gut microbiota, these findings are often observational, lack long-term follow-up, and are complicated by confounding factors. Therefore, further research is urgently needed to clarify the long-term effects of NSS based on the type, quantity, and age at first exposure, especially regarding links to diabetes, obesity, and other health conditions.

Global health guidelines now recommend limiting NSS intake in healthy populations, especially young children. In paediatric cases, NSS should be reserved for diabetes management or when other obesity prevention strategies are insufficient. Regulatory action should ensure clear communication to reduce confusion among consumers and health professionals.

Within the EU, a lack of clear labelling on NSS-containing foods has been identified. Evidence from Mexico’s successful warning-label policy suggests that such measures can reduce NSS consumption and help parents make healthier dietary choices for their children. These insights should inform future international policies and strategies.

Health strategies must focus on reducing children’s preference for sweet tastes by promoting water over sweetened beverages, including those with NSS. Relying on highly sweet alternatives may hinder children’s sensory development, reinforce sweet food preferences, and negatively affect overall dietary quality. Establishing healthy eating habits early in life is key to preventing chronic diet-related diseases.

Given the rapid development occurring in early childhood and limited data on the safety of NSS in this age group, a precautionary approach is warranted. Healthcare professionals, including paediatricians, nutritionists, pharmacists, and nurses, must have clear, evidence-based guidance on NSS to provide effective counselling, contributing to the prevention and management of NCDs in children.

## Data Availability

The original contributions presented in the study are included in the article/supplementary material, further inquiries can be directed to the corresponding author.

## References

[1] WHO. (2025). Available online at: https://www.who.int/data/gho/data/themes/sustainable-development-goals (Accessed July 25, 2025).

[2] ESPEN. (2025). Available online at: https://www.espen.org/ (Accessed July 25, 2025).

[3] WHO World health statistics Geneva WHO; (2025) 1–88

[4] United Nations Children’s Fund (UNICEF), World Health Organization (WHO), International Bank for Reconstruction and Development/The World Bank. Levels and trends in child malnutrition: key finding of the 2023 edition. Levels and trends in child malnutrition: UNICEF / WHO / World Bank Group Joint Child Malnutrition Estimates: Key findings of the 2023 edition. New York: UNICEF and WHO (2023).

[5] WHO. Zagreb declaration. Zagreb, Croatia, pp. 1–3. (2023). Available online at: https://apps.who.int/iris/handle/10665/353747 (Accessed July 25, 2025).

[6] International Diabetes Federation (IDF). IDF Diabetes Atlas 11th edn. Brussels, Belgium: International Diabetes Federation. pp. 1–161 (2025). Available online at: https://diabetesatlas.org/resources/idf-diabetes-atlas-2025/ (Accessed July 25, 2025).

[7] WHO. Guideline: Sugars intake for adults and children. Geneva, Switzerland: WHO (2015). 25905159

[8] Turck D Bohn T Castenmiller J de Henauw S Hirsch-Ernst KI Knutsen HK . Tolerable upper intake level for dietary sugars. EFSA J. (2022) 20:e07074. doi: 10.2903/j.efsa.2022.7074, PMID: 35251356 PMC8884083

[9] Walton J Wittekind A. Soft drink intake in Europe—a review of data from nationally representative food consumption surveys. Nutrients. (2023) 15:1368. doi: 10.3390/nu15061368, PMID: 36986099 PMC10051353

[10] Russell C Baker P Grimes C Lindberg R Lawrence MA. Global trends in added sugars and non-nutritive sweetener use in the packaged food supply: drivers and implications for public health. Public Health Nutr. (2023) 26:952–64. doi: 10.1017/S1368980022001598, PMID: 35899782 PMC10346066

[11] OECD. OECD-FAO Agricultural Outlook 2023–2032. Paris: OECD Publishing (2023).

[12] Kossiva L Kakleas K Christodouli F Soldatou A Karanasios S Karavanaki K. Chronic use of artificial sweeteners: pros and cons. Nutrients. (2024) 16:3162. doi: 10.3390/nu16183162, PMID: 39339762 PMC11435027

[13] Liu Q Wang M Hou Y Chen R Liu H Han T . Deciphering the multifaceted effects of artificial sweeteners on body health and metabolic functions: a comprehensive review and future perspectives. Crit Rev Food Sci Nutr. (2024) 65:5394–416. doi: 10.1080/10408398.2024.2411410, PMID: 39368060

[14] Singh S Kohli A Trivedi S Kanagala SG Anamika FNU Garg N . The contentious relationship between artificial sweeteners and cardiovascular health. Egypt J Intern Med. (2023) 35:43. doi: 10.1186/s43162-023-00232-1

[15] Baker-Smith CM De Ferranti SD Cochran WJ. The use of nonnutritive sweeteners in children. Pediatrics. (2019) 144:1–18. doi: 10.1542/peds.2019-2765, PMID: 31659005

[16] Hedrick VE Nieto C Grilo MF Sylvetsky AC. Non-sugar sweeteners: helpful or harmful? The challenge of developing intake recommendations with the available research. BMJ. (2023) 383:e075293. doi: 10.1136/bmj-2023-075293, PMID: 37813435 PMC11426959

[17] IARC. Summary of findings of the evaluation of aspartame at the International Agency for Research on Cancer (IARC) Monographs Programme’s 134th meeting, 6–13 June 2023 and the JOINT FAO/WHO EXPERT COMMITTEE ON FOOD ADDITIVES (JECFA) 96th meeting, 27 June–6 July. pp. 1–10 (2023). Available online at: https://cdn.who.int/media/docs/default-source/nutrition-and-food-safety/jecfa-summary-of-findings-aspartame.pdf?sfvrsn=a531e2c1_22&download=true (Accessed July 25, 2025).

[18] WHO. Use of non-sugar sweeteners: WHO guideline summary. Geneva: World Health Organization. 37256996

[19] Khan TA Lee JJ Ayoub-Charette S Noronha JC McGlynn N Chiavaroli L . WHO guideline on the use of non-sugar sweeteners: a need for reconsideration. Eur J Clin Nutr. (2023) 77:1009–13. doi: 10.1038/s41430-023-01314-7, PMID: 37723261 PMC10630128

[20] Imamura F O’Connor L Ye Z Mursu J Hayashino Y Bhupathiraju SN . Consumption of sugar sweetened beverages, artificially sweetened beverages, and fruit juice and incidence of type 2 diabetes: systematic review, meta-analysis, and estimation of population attributable fraction. BMJ. (2015) 351:1–12. doi: 10.1136/bmj.h3576 PMC4510779 26199070

[21] European Commission. Regulation (EC) no 178/2002 of the European Parliament and of the council of 28 January 2002 laying down the general principles and requirements of food law, establishing the European Food Safety Authority and laying down procedures in matters of food saf. Off J Eur Communities. (2002) L31:1–24.

[22] EC. Implementation of directive 1989/398/EEC, relating to foodstuffs intended for particular nutritional uses, pp. 27–32 (1989).

[23] EC. Regulation (EC) no 1333/2008 of the European Parliament ans of the Council of 16 December 2998 on food additives. Off J Eur Union. (2008) L356:16–33.

[24] European Commission. Food-based dietary guidelines – guidance on sweeteners. (2025). Available online at: https://knowledge4policy.ec.europa.eu/health/food-guidelines-table-24_en (Accessed July 7, 2025)

[25] SWEET. Sweeteners and sweetness enhancers: impact on health, obesity, safety and sustainability. CORDIS. (2024). doi: 10.3030/774293

[26] Fidler Mis N Braegger C Bronsky J Campoy C Domellöf M Embleton ND . Sugar in infants, children and adolescents: a position paper of the European Society for Paediatric Gastroenterology, Hepatology and Nutrition Committee on Nutrition. J Pediatr Gastroenterol Nutr. (2017) 65:681–96. doi: 10.1097/MPG.0000000000001733, PMID: 28922262

[27] Dereń K Weghuber D Caroli M Koletzko B Thivel D Frelut ML . Consumption of sugar-sweetened beverages in paediatric age: a position paper of the European Academy of Paediatrics and the European Childhood Obesity Group. Ann Nutr Metab. (2019) 74:296–302. doi: 10.1159/000499828, PMID: 31013493

[28] Health Canada. *Canada’s dietary guidelines for health professionals and policy makers*, p. 62. (2019). Available online at: https://food-guide.canada.ca/sites/default/files/artifact-pdf/CanadasDietaryGuidelines.pdf (Accessed July 25, 2025).

[29] Government of Canada. Nutrition for healthy term infants: Recommendations from six to 24 months. The infant feeding guidelines are under revision (2024) Available online at: https://www.canada.ca/en/health-canada/services/canada-food-guide/resources/nutrition-healthy-term-infants/nutrition-healthy-term-infants-recommendations-birth-six-months/6-24-months.html (Accessed July 7, 2025)

[30] USDA. Dietary guidelines for Americans. 9th ed. Washington, D.C., USA: U.S. Department of Agriculture (USDA).(2020). 1–164. doi: 10.1093/ajcn/34.1.121

[31] Ryan DH Kahan S. Guideline recommendations for obesity management. Med Clin North Am. (2018) 102:49–63. doi: 10.1016/j.mcna.2017.08.006, PMID: 29156187

[32] Grilo MF Taillie LS Sylvetsky AC. The widespread presence of non-nutritive sweeteners challenges adherence to beverage guidance for children. Front Public Health. (2023) 11:8–11. doi: 10.3389/fpubh.2023.1221764, PMID: 37663855 PMC10472131

[33] HER. Consensus statement healthy beverage consumption in early childhood recommendations from key National Health and Nutrition Organizations. (2019). Available online at: http://healthyeatingresearch.org (Accessed July 7, 2025)

[34] Vidal L Bove I Brunet G Girona A Alcaire F Antúnez L . Are the recommendations of pediatricians about complementary feeding aligned with current guidelines in Uruguay? Public Health Nutr. (2021) 24:641–50. doi: 10.1017/S1368980020005352, PMID: 33413708 PMC11574833

[35] EFSA. Overview of the procedures currently used at EFSA for the assessment of dietary exposure to different chemical substances. EFSA J. (2011) 9:1–33. doi: 10.2903/j.efsa.2011.2490 PMC13093108 42015991

[36] Martyn D Darch M Roberts A Lee HY Tian TY Kaburagi N . Low-/no-calorie sweeteners: a review of global intakes. Nutrients. (2018) 10:1–39. doi: 10.3390/nu10030357 PMC5872775 29543782

[37] Silva PD Cruz R Casal S. Sugars and artificial sweeteners in soft drinks: a decade of evolution in Portugal. Food Control. (2021) 120:107481. doi: 10.1016/j.foodcont.2020.107481

[38] BfR. Sugar alternatives: how much sweetener is there in soft drinks? BfR-Stellungnahmen. (2023) 6:1–18. doi: 10.17590/20230414-150752-0

[39] Seesen M Chang K Parnham JC Laverty AA Millett C Rauber F . Association of low-calorie sweetened product consumption and intakes of free sugar and ultra-processed foods in UK children: a national study from 2008 to 2019. Eur J Nutr. (2025) 64:230. doi: 10.1007/s00394-025-03740-8, PMID: 40569454 PMC12202694

[40] Sambra V López-Arana S Cáceres P Abrigo K Collinao J Espinoza A . Overuse of non-caloric sweeteners in foods and beverages in Chile: a threat to consumers’ free choice? Front Nutr. (2020) 7:1–8. doi: 10.3389/fnut.2020.00068, PMID: 32626722 PMC7311776

[41] Zancheta Ricardo C Corvalán C Smith Taillie L Quitral V Reyes M. Changes in the use of non-nutritive sweeteners in the Chilean food and beverage supply after the implementation of the food labeling and advertising law. Front Nutr. (2021) 8:1–10. doi: 10.3389/fnut.2021.773450 PMC8630583 34859036

[42] Reyes M Pino C Ortega A Pemjean I Corvalán C Garmendia ML. Potential actions for preventing high consumption of non-nutritive sweeteners among Chilean children and adolescents: recommendations from a panel of relevant actors. Public Health Nutr. (2024) 27:e199–12. doi: 10.1017/S1368980024001745, PMID: 39370955 PMC11505000

[43] European Commission (EC). Health promotion and disease prevention knowledge gateway. Available online at: https://knowledge4policy.ec.europa.eu/health-promotion-knowledge-gateway/sugars-sweeteners-11_en?utm_source=chatgpt.com (Accessed September 2, 2025).

[44] Rebolledo N Bercholz M Adair L Corvalán C Ng SW Taillie LS. Sweetener purchases in Chile before and after implementing a policy for food Labeling, marketing, and sales in schools. Curr Dev Nutr. (2023) 7:100016. doi: 10.1016/j.cdnut.2022.100016, PMID: 37180088 PMC10111599

[45] Salgado JC Pedraza LS Contreras-Manzano A Aburto TC Tolentino-Mayo L Barquera S. Product reformulation in non-alcoholic beverages and foods after the implementation of front-of-pack warning labels in Mexico. PLoS Med. (2025) 22:e1004533–25. doi: 10.1371/journal.pmed.1004533, PMID: 40100880 PMC11918434

[46] EC. Health promotion and disease prevention knowledge gateway. (2021). Available online at: https://knowledge4policy.ec.europa.eu/health-promotion-knowledge-gateway/sugars-sweeteners-11_en?utm_source=chatgpt.com (Accessed July 7, 2025)

[47] Swedish Food Agency. The keyhole design manual. Sweden: Swedish Food Agency. (2021). 1–21.

[48] Storcksdieck Genannt Bonsmann S. Comprehensive mapping of national school food policies across the European Union plus Norway and Switzerland. Nutr Bull. (2014) 39:369–73. doi: 10.1111/nbu.12109, PMID: 25663818 PMC4314700

[49] Micha R Karageorgou D Bakogianni I Trichia E Whitsel LP Story M . Effectiveness of school food environment policies on children’s dietary behaviors: a systematic review and meta-analysis. PLoS One. (2018) 13:e0194555. doi: 10.1371/journal.pone.0194555, PMID: 29596440 PMC5875768

[50] UNC. The global food research program: Sugary drink taxes around the world. (2021). Available online at: https://globalfoodresearchprogram.org/wp-content/uploads/2020/08/SugaryDrink_tax_maps_2020_August_REV.pdf (Accessed July 25, 2025).

[51] Andreyeva T Marple K Marinello S Moore TE Powell LM. Outcomes following taxation of sugar-sweetened beverages. JAMA Netw Open. (2022) 5:e2215276. doi: 10.1001/jamanetworkopen.2022.15276, PMID: 35648398 PMC9161017

[52] Wierzejska RE Wiosetek-Reske A Wojda B. Perceptions of young people regarding the tax on sweetened drinks in Poland and an assessment of changes in their consumption in terms of taxation. Beverages. (2024) 10:030085. doi: 10.3390/beverages10030085

[53] Martínez J Mart J. Heterogeneous effects of SSB taxes: evidence from Spain, pp. 1–36 (2023). Available online at: https://www.esade.edu/ecpol/wp-content/uploads/2022/10/EcPol_Bebidas-Azucaradas_v1.pdf (Accessed July 25, 2025).

[54] Smith E Scarborough P Rayner M Briggs ADM. Should we tax unhealthy food and drink? Proc Nutr Soc. (2018) 77:314–20. doi: 10.1017/S0029665117004165, PMID: 29332613 PMC5912513

[55] Driessen C Kelly B Sing F Backholer K. Parents’ perceptions of children’s exposure to unhealthy food marketing: a narrative review of the literature. Curr Nutr Rep. (2022) 11:9–18. doi: 10.1007/s13668-021-00390-0, PMID: 35278205 PMC8942884

[56] Jensen ML Choi YY Fleming-Milici F Harris JL. Caregivers’ understanding of ingredients in drinks served to young children: opportunities for nutrition education and improved labeling. Curr Dev Nutr. (2022) 6:nzab151–10. doi: 10.1093/cdn/nzab151, PMID: 35047722 PMC8760421

